# Supplementary material for: Circulating PACAP levels are associated with increased amygdala-default mode network resting-state connectivity in posttraumatic stress disorder
Source: Neuropsychopharmacology. 2023 May 9;48(8):1245–54. doi: 10.1038/s41386-023-01593-5 (PMC10267202; doi:10.1038/s41386-023-01593-5)
Supplement: Supplementary file 1 — Supplemental Material [file 41386_2023_1593_MOESM1_ESM.pdf]

SUPPLEMENTAL MATERIALS

MATERIALS AND METHODS

Participants

Inclusion criteria included ability to provide written informed consent, 18-55 years old, and any gender. Given our interest in sex differences, specifically the influence of estrogen modulation of PACAP, participants were required to be the same sex as assigned at birth, female subjects were to be premenopausal, and participants with a history of receiving hormonal replacement therapy or undergoing surgery to change biological sex were excluded. Other exclusion criteria included left-handedness, medical conditions that would confound results, such as a seizure or other neurological disorder, inability to tolerate blood draws, history of moderate to severe traumatic brain injury, current treatment with an antipsychotic (unless prescribed only for sleep), MR contraindications, including metal implants and claustrophobia, positive pregnancy test for female participants on the day of scanning. In addition, participants were excluded for lifetime history of schizophrenia or schizoaffective disorder and if they met for current (past month) moderate-to-severe alcohol or substance use disorder, psychotic disorder, anorexia, obsessive compulsive disorder, or manic or mixed mood episode.

PACAP Assays

Participants were instructed not to eat the morning of their visit prior to their blood draw. Samples were centrifuged at 3500 rpm for 15 minutes. Plasma was extracted and stored at -80 °C until analysis. Optimal sample volume was determined in dilution tests, and all values represent the mean from assay duplicates; intra-assay variation was approximately 9%. Assay midpoint was 1.1 fmol and detection limit from the linear range of the standard curve was 0.2 fmol.

MRI data acquisition and preprocessing

T1-weighted 3D MPRAGE structural images were initially acquired using the HCP 0.7mm resolution sequence (TR/TE: 2400/2.14 ms; flip angle: 8 deg; FOV: 224 x 224; voxel size: 0.7mm isotropic), and eyes-open resting state T2-weighted echoplanar images were initially acquired using the HCP Young Adult sequence (TR/TE: 720/33.1 ms, in-plane resolution: 2mm; voxels: 2mm isotropic; multiband factor = 8; two runs of 600 frames each). Early in the study, imaging protocols were transitioned to the HCP Lifespan protocol (n = 78) – T1-weighted 3D MPRAGE structural images were acquired using the HCP 0.8mm resolution sequence (TR/TEs: 2500/1.81/3.6/5.39/7.18; flip angle: 8 deg; FOV: 256 x 240; voxel size: 0.8mm isotropic), and eyes-open resting state T2-weighted echoplanar images were acquired using the HCP Lifespan sequence (TR/TE: 800/37 ms, in-plane resolution: 2mm; voxels: 2mm isotropic; multiband factor = 8; anterior-posterior phase encoding; two runs of 488 frames each).

T1-weighted (T1w) images were corrected for intensity non-uniformity [1]. Brain tissue segmentation of cerebrospinal fluid (CSF), white matter (WM) and gray matter (GM) was performed on the brain-extracted T1w [2]. Brain surfaces were reconstructed using recon-all [3], and the brain mask estimated previously was refined with a custom variation of the method to reconcile ANTs-derived and FreeSurfer-derived

segmentations of the cortical GM of Mindboggle [4]. Volume-based spatial normalization to MNI standard space (MNI152NLin6Asym) was performed through nonlinear registration with antsRegistration (ANTs 2.3.3), using brain-extracted versions of both T1w reference and the T1w template.

EPI images were corrected for susceptibility distortions using the fMRIPrep fieldmap-less approach [5]. Based on the estimated susceptibility distortion, a corrected EPI (echo-planar imaging) reference was calculated for a more accurate co-registration with the anatomical reference. The reference was co-registered to the T1w reference with six degrees of freedom [6]. Head motion parameters with respect to the reference (transformation matrices, and six corresponding rotation and translation parameters) were estimated before any spatiotemporal filtering [7]. EPI images were slice-time corrected [8]. The time series were resampled onto their original, native space by applying a single, composite transform to correct for head motion and susceptibility distortions. The time series were resampled into standard space, generating a preprocessed run in MNI152NLin6Asym space. First, a reference volume and its skull-stripped version were generated using a custom methodology of fMRIPrep. Automatic removal of motion artifacts using independent component analysis (ICA-AROMA) [9] was performed on the preprocessed images on MNI space time-series after removal of non-steady state volumes and spatial smoothing with an isotropic, Gaussian kernel of 6mm FWHM (full-width half-maximum). Corresponding "non-aggressively" denoised runs were produced after such smoothing [9].

RESULTS

Sex-differences in circulating PACAP levels

Female participants demonstrated significantly greater circulating PACAP levels than males ($t = 5.34$, $p < 0.001$). These differences were robust to controlling for other clinical and demographic variables demonstrating sex differences (age and total PTSD symptom severity; $F = 14.60$, $p < 0.001$), suggesting sex-related differences in circulating PACAP levels were not attributable to other differences.

No effect of scanner protocol

The results were robust to controlling for scanner protocol. Circulating PACAP levels were associated with rAMYG-PCC/Precun (semi-partial $r = 0.25$, $p = 0.011$) and rAMYG-lANG connectivity ($sr = 0.30$, $p = 0.004$). Sex-specific effects similarly held – in women, circulating PACAP levels were associated with rAMYG-PCC/Precun ($sr = 0.30$, $p = 0.014$) and rAMYG-lANG connectivity ($sr = 0.34$, $p = 0.005$). No effects emerged for men (p's > 0.364). Scanning protocols did not differ in any of the AMYG FC values (p's > 0.268).

Lifespan protocol effects

ROI-based functional connectivity analyses revealed a positive association between circulating PACAP levels and rAMYG-PCC/Precun connectivity ($r = 0.30$, $p = 0.009$, FDR $p < 0.05$) and rAMYG-lANG connectivity ($r = 0.36$, $p = 0.002$, FDR $p < 0.05$), but not rAMYG-mPFC ($r = 0.03$, $p = 0.796$) or rAMYG-rANG ($r = 0.19$, $p = 0.099$). No effects were seen with the lAMYG (p's > 0.298).

Planned analyses of sex-specific effects revealed PACAP associations with rAMYG-PCC/Precun and rAMYG-lANG connectivity were present only in female participants (PCC/Precun: $r = 0.38$, $p = 0.004$, FDR $p < 0.05$; lANG: $r = 0.40$, $p = 0.002$, FDR $p < 0.05$). No associations were seen in male participants (PCC/Precun: $r = -0.22$, $p = 0.378$; lANG: $r = 0.06$ $p = 0.807$).

Planned analyses of AMYG subregion-specific effects suggested associations with PACAP were somewhat unique to the CMA. PACAP was associated with CMA-PCC/Precun connectivity ($r = 0.25$, $p = 0.031$) but not BLA-PCC/Precun connectivity ($r = 0.16$, $p = 0.171$). No such specificity was seen for the lANG (CMA $r = 0.22$, $p = 0.058$; BLA $r = 0.23$, $p = 0.052$).

Subregion effects with PCC/Precun connectivity were similarly specific to females (CMA: $r = 0.34$, $p = 0.010$; BLA: $r = 0.24$, $p = 0.078$) and not males (CMA: $r = -0.11$, $p = 0.670$; BLA: $r = -0.33$, $p = 0.172$). Whole-brain analyses again confirmed the spatial specificity of this CMA-effect in females to a bilateral PCC/Precun cluster (**Figure 3B**, $k = 201$, cluster FDR $q = 0.021$, peak = 10, -54, 34, $T = 4.06$), with no such effects emerging with the BLA.

Controlling for demographic and clinical variables
The results were robust to controlling for age, sex, PTSD diagnosis, and total life-time trauma exposure (LEC-5) as covariates (sr = semi-partial r). Circulating PACAP levels were associated with rAMYG-PCC/Precun ($sr = 0.25$, $p = 0.023$) and rAMYG-lANG connectivity ($sr = 0.30$, $p = 0.005$). Sex-specific effects were similarly robust to age, PTSD diagnosis, and total trauma exposure – in women, circulating PACAP levels were associated with rAMYG-PCC/Precun ($sr = 0.32$, $p = 0.008$) and rAMYG-lANG connectivity ($sr = 0.35$, $p = 0.004$), while no effects emerged for men (p’s > 0.478).

Whole-brain results
Whole-brain associations with circulating PACAP levels, including clusters that did not survive correction for multiple comparisons, are provided for the rAMYG seed for the full sample (Table S1) and female participants (Table S2), as well as for the rCMA (Table S3) and rBLA seeds (Table S4).

Supplementary Figures

Figure S1

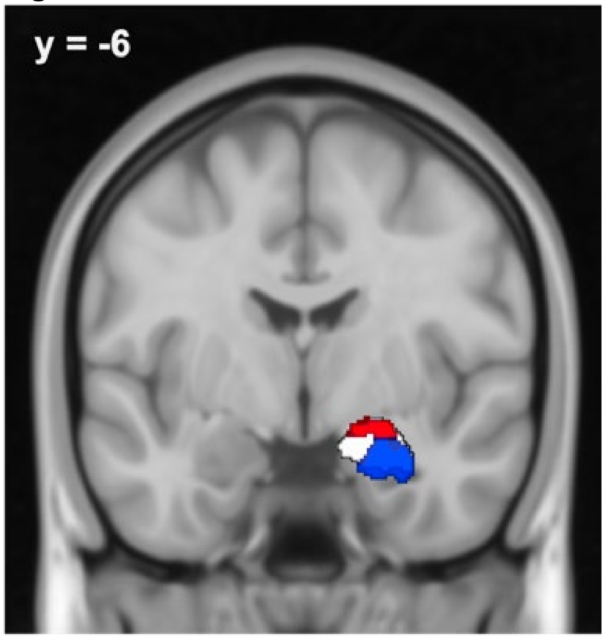

<u>Figure S1.</u> Amygdala subregion ROIs. JuBrain Atlas centromedial (CMA, red) and basolateral (BLA, blue) amygdala subregion ROIs [53] overlaid on the canonical Harvard-Oxford amygdala ROI (white) [50]. Only the right hemisphere is depicted.

Table S1. Whole-brain rAMYG seed-based connectivity with circulating PACAP levels. p < 0.005 (uncorrected height threshold), k = 50 arbitrary cluster threshold.

Statistics: p-values adjusted for search volume									
==									
cluster	cluster	cluster	cluster	peak	peak	peak	peak	peak	
p(FWE-corr)	p(FDR-corr)	equivk	p(unc)	p(FWE-corr)	p(FDR-corr)	T	equivZ	p(unc)	x,y,z {mm}
--									
0.005	0.006	278	0	0.89	0.758	4.29	4.08	0	-4 -68 26
				0.899	0.758	4.28	4.06	0	10 -54 32
				1	0.758	3.65	3.51	0	-12 -60 16
0.279	0.091	125	0.003	0.936	0.758	4.22	4.01	0	-30 0 50
				1	0.862	3.08	2.99	0.001	-20 0 44
0.012	0.006	245	0	1	0.758	3.83	3.67	0	-38 -82 26
				1	0.758	3.66	3.51	0	-44 -68 26
				1	0.789	3.29	3.18	0.001	-44 -78 34
0.431	0.118	108	0.005	1	0.758	3.78	3.63	0	-16 36 38
				1	0.758	3.44	3.32	0	-24 32 40
0.294	0.091	123	0.003	1	0.758	3.71	3.56	0	-26 -70 48
				1	0.758	3.53	3.4	0	-26 -82 44
				1	0.978	2.73	2.66	0.004	-18 -82 50

Table S2. Whole-brain rAMYG seed-based connectivity with circulating PACAP levels in female participants. p < 0.005 (uncorrected height threshold), k = 50 arbitrary cluster threshold.

Statistics: p-values adjusted for search volume									
==									
cluster	cluster	cluster	cluster	peak	peak	peak	peak	peak	
p(FWE-corr)	p(FDR-corr)	equivk	p(unc)	p(FWE-corr)	p(FDR-corr)	T	equivZ	p(unc)	x,y,z {mm}
--									
0.719	0.356	81	0.01	0.861	0.994	4.43	4.13	0	-26 4 46
0.002	0.001	296	0	0.986	0.994	4.15	3.9	0	-52 -68 24
				0.993	0.994	4.1	3.86	0	-40 -82 32
				1	0.994	3.35	3.21	0.001	-38 -62 24
0.001	0.001	319	0	0.994	0.994	4.09	3.85	0	12 -54 32
				0.996	0.994	4.06	3.82	0	-6 -66 26
				1	0.994	3.83	3.63	0	-10 -58 18
0.809	0.358	74	0.014	1	0.994	3.41	3.26	0.001	34 4 60
				1	0.994	3.29	3.16	0.001	30 2 50
0.732	0.356	80	0.011	1	0.994	3.29	3.16	0.001	-26 -72 48
				1	0.994	3.29	3.16	0.001	-26 -82 44
				1	0.994	3.06	2.95	0.002	-28 -62 50

Table S3. Whole-brain rCMA seed-based connectivity with circulating PACAP levels in female participants. $p < 0.005$ (uncorrected height threshold), k = 50 arbitrary cluster threshold.

Statistics: p-values adjusted for search volume

cluster p(FWE-corr)	cluster p(FDR-corr)	cluster equivk	cluster p(unc)	peak p(FWE-corr)	peak p(FDR-corr)	peak T	peak equivZ	peak p(unc)	x,y,z {mm}
0.023	0.021	201	0	0.997	0.987	4.06	3.83	0	10 -54 34
				1	0.987	3.94	3.73	0	0 -62 30
				1	0.987	3.65	3.47	0	-6 -66 26
0.754	0.629	76	0.011	0.997	0.987	4.05	3.82	0	-6 -18 -12
				1	0.987	3.81	3.61	0	-4 -22 -22
0.97	0.778	54	0.028	0.999	0.987	4	3.77	0	-28 6 44
				1	0.987	2.93	2.83	0.002	-26 -2 42
0.985	0.778	50	0.033	0.999	0.987	3.99	3.77	0	-28 30 40
				1	0.987	3.59	3.42	0	-22 26 32

Table S4. Whole-brain rBLA seed-based connectivity with circulating PACAP levels in female participants. $p < 0.005$ (uncorrected height threshold), k = 50 arbitrary cluster threshold.

Statistics: p-values adjusted for search volume

cluster p(FWE-corr)	cluster p(FDR-corr)	cluster equivk	cluster p(unc)	peak p(FWE-corr)	peak p(FDR-corr)	peak T	peak equivZ	peak p(unc)	x,y,z {mm}
0.969	0.777	54	0.027	0.22	0.274	5.11	4.68	0	22 -20 58
0.051	0.048	172	0	0.999	0.909	4.01	3.78	0	20 -4 52
				1	0.909	3.59	3.42	0	12 -6 54
				1	0.909	3.48	3.32	0	30 -2 58
0.723	0.581	78	0.01	1	0.909	3.8	3.6	0	-20 0 46
				1	0.909	3.68	3.5	0	-30 0 50
				1	0.971	3.21	3.09	0.001	-26 -6 44

Table S5. Whole-brain rAMYG seed-based connectivity with CAPS-Anxious Arousal symptom cluster severity. $p < 0.005$ (uncorrected height threshold), k = 50 arbitrary cluster threshold.

Statistics: p-values adjusted for search volume

cluster p(FWE-corr)	cluster p(FDR-corr)	cluster equivk	cluster p(unc)	peak p(FWE-corr)	peak p(FDR-corr)	peak T	peak equivZ	peak p(unc)	x,y,z {mm}
0.188	0.082	138	0.002	0.998	0.981	3.93	3.76	0	-6 -26 32
				1	0.981	3.86	3.7	0	-4 -34 24
				1	0.981	2.9	2.83	0.002	2 -14 32
0.108	0.082	158	0.001	1	0.981	3.78	3.63	0	12 -60 40
				1	0.981	3.46	3.34	0	2 -56 50
				1	0.981	3.3	3.2	0.001	8 -68 44
0.984	0.789	53	0.035	1	0.981	3.52	3.4	0	48 4 -30
				1	0.981	3.33	3.23	0.001	54 -6 -34

Table S6. Whole-brain rAMYG seed-based connectivity with CAPS-Startle Response symptom severity. $p < 0.005$ (uncorrected height threshold), $k = 50$ arbitrary cluster threshold.

Statistics: p-values adjusted for search volume									
==									
cluster	cluster	cluster	cluster	peak	peak	peak	peak	peak	
p(FWE-corr)	p(FDR-corr)	equivk	p(unc)	p(FWE-corr)	p(FDR-corr)	T	equivZ	p(unc)	x,y,z {mm}
--									
0.698	0.384	85	0.01	0.74	0.995	4.45	4.22	0	-6 -38 46
0.022	0.021	218	0	0.976	0.995	4.11	3.93	0	-126
				1	0.995	3.1	3.01	0.001	-28 -84 8
				1	0.995	2.98	2.9	0.002	-116
0.928	0.549	64	0.023	1	0.995	3.76	3.61	0	-4 -34 24
0.291	0.166	122	0.003	1	0.995	3.7	3.56	0	6 -62 22
				1	0.995	3.37	3.26	0.001	4 -62 34
				1	0.995	3.13	3.04	0.001	6 -68 42
0.942	0.549	62	0.025	1	0.995	3.7	3.56	0	36 -84 2

SUPPLEMENTAL REFERENCES

1. Tustison NJ, Avants BB, Cook PA, Zheng Y, Egan A, Yushkevich PA, et al. N4ITK: improved N3 bias correction. IEEE Trans Med Imaging. 2010;29:1310–1320.
2. Zhang Y, Brady M, Smith S. Segmentation of brain MR images through a hidden Markov random field model and the expectation-maximization algorithm. IEEE Trans Med Imaging. 2001;20:45–57.
3. Dale AM, Fischl B, Sereno MI. Cortical surface-based analysis. I. Segmentation and surface reconstruction. NeuroImage. 1999;9:179–194.
4. Klein A, Ghosh SS, Bao FS, Giard J, Häme Y, Stavsky E, et al. Mindboggling morphometry of human brains. PLoS Comput Biol. 2017;13:e1005350.
5. Wang S, Peterson DJ, Gatenby JC, Li W, Grabowski TJ, Madhyastha TM. Evaluation of Field Map and Nonlinear Registration Methods for Correction of Susceptibility Artifacts in Diffusion MRI. Front Neuroinformatics. 2017;11:17.
6. Greve DN, Fischl B. Accurate and robust brain image alignment using boundary-based registration. NeuroImage. 2009;48:63–72.
7. Jenkinson M, Bannister P, Brady M, Smith S. Improved optimization for the robust and accurate linear registration and motion correction of brain images. NeuroImage. 2002;17:825–841.
8. Cox RW, Hyde JS. Software tools for analysis and visualization of fMRI data. NMR Biomed. 1997;10:171–178.
9. Pruim RHR, Mennes M, van Rooij D, Llera A, Buitelaar JK, Beckmann CF. ICA-AROMA: A robust ICA-based strategy for removing motion artifacts from fMRI data. NeuroImage. 2015;112:267–277.
